# The Use of Tricyclo-DNA Oligomers for the Treatment of Genetic Disorders

**DOI:** 10.3390/biomedicines6010002

**Published:** 2017-12-22

**Authors:** Philippine Aupy, Lucía Echevarría, Karima Relizani, Aurélie Goyenvalle

**Affiliations:** 1 INSERM U1179, UFR des Sciences de la Santé, University of Versailles St-Quentin, 78180 Montigny le Bretonneux, France; philippine.aupy2@uvsq.fr (P.A.); lucia.echevarria-zamora@uvsq.fr (L.E.); sonia.relizani-karima@uvsq.fr (K.R.); 2 SQY Therapeutics, University of Versailles St-Quentin, 78180 Montigny le Bretonneux, France

**Keywords:** antisense oligonucleotides, tricyclo-DNA, gapmers, splice-switching, exon-skipping, exon-re-inclusion, delivery

## Abstract

Antisense Oligonucleotides (ASOs) represent very attractive therapeutic compounds for the treatment of numerous diseases. The antisense field has remarkably progressed over the last few years with the approval of the first antisense drugs and with promising developments of more potent and nuclease resistant chemistries. Despite these recent clinical successes and advances in chemistry and design, effective delivery of ASOs to their target tissues remains a major issue. This review will describe the latest advances obtained with the tricyclo-DNA (tcDNA) chemistry which displays unique pharmacological properties and unprecedented uptake in many tissues after systemic administration. We will examine the variety of therapeutic approaches using both fully modified tcDNA-ASOs and gapmers, including splice switching applications, correction of aberrant splicing, steric blocking strategies and targeted gene knock-down mediated by RNase H recruitment. We will then discuss the merits and potential liabilities of the tcDNA chemistry in the context of ASO drug development.

## 1. Introduction

Antisense oligonucleotides (ASOs) are single-stranded DNA sequences able to modify mRNA by increasing or decreasing gene expression or modulate splicing. ASOs are typically 13–22 nucleotides, while shorter and longer sequences are also tested [1,2,3]. Hybridization of ASOs to their target is made by Watson-Crick base pairing although the exact mechanism by which ASOs find their target requires further elucidation [4,5].

ASOs were first used in 1978 by Zamecnik and Stephenson as an approach for sequence-specific silencing of gene expression [6]. Since then, antisense technology have rapidly evolved making them a valuable tool for human therapeutics [7].

First unmodified ASOs were composed of a phosphodiester backbone and sugar rings but they were rapidly degraded within cells and blood [8]. Since then, various modifications have been introduced in their chemical structure in order to protect them from nuclease activity and increase their stability and affinity to target RNA. Natural phosphodiester bond is highly sensitive to nucleases and has therefore been the first site for chemical modification. Typical phosphorothioate (PS) backbones, in which one of the non-bridging phosphate O-atoms is replaced with a S-atom [9], is still one of the most largely adopted variation for antisense drugs. However they are known to cause undesirable effects mainly due to their capacity to bind plasma proteins [10].

Second generation ASOs included chemical modification in the 2′-position of the sugar moiety such as 2′-*O*-methyl (2′OME), 2′-*O*-methoxyethyl (2′MOE), 2′-fluorinated (2′F) and 2′-*O*-aminopropyl analogues which considerably increased RNA binding affinity and pharmacokinetics compared to their 2′-deoxy counterpart [11]. In addition, 2′-modified oligonucleotides were reported to reduce immune stimulation side effect compared to PS-modified ASOs [12]. Chemical ASO modification have kept evolving and, new generation of molecules include a wide variety of designs such as morpholinos [13], locked nucleic acids (LNAs) [14], 2′,4′-constrained ethyl (cEt) [15] or peptide nucleic acids (PNAs) [16]. It is important to note that these modified ASOs became unable to elicit RNAse H mediated RNA degradation, hence additional strategies involving mixed oligonucleotides with a DNA core flanked by 2′ modified nucleotides known as gapmers have been developed as it will be discussed later.

Numerous ASO drugs are currently being tested in clinical trials (see ClinicalTrials.gov) and four of them have received marketing approval so far. Two of these compounds work via RNAse H mediated cleavage of RNA (Fomivirsen and Mipomersen from IONIS Pharmaceuticals) and the other two aim at modulating splicing, also known as splice switching oligonucleotides (SSO) (Eteplirsen from Sarepta and Nusinersen from Biogen) [17,18].

These recent successes elicit optimism and hope for the development of future therapeutics using the antisense technology. However, clinical benefit of ASO based therapy is still very limited because of the relatively poor delivery to the target tissues. It is assumed that only about 1% of ASO drug reach the correct cellular compartment and the majority of them are not able to cross the blood-brain barrier (BBB) to target the central nervous system (CNS). Delivery therefore remains one of the key issue in the antisense field and recent reviews have nicely covered these challenges [18,19]. Further efforts are thus needed in order to develop new approaches to improve effectiveness of ASOs and be able to benefit from the clinical use of ASOs technology.

**Figure 1:**
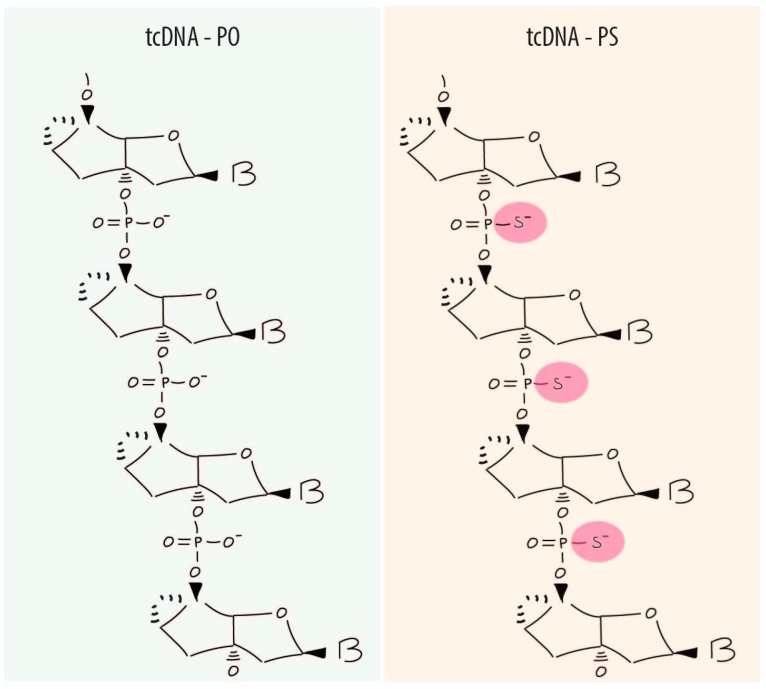
Antisense tcDNA molecule with natural phospodiester (PO) (left panel) or modified phosphorothioate (PS) (right panel) backbones (B = base adenine, guanine, cytosine or thymidine).

In the late nineties, a new generation of oligonucleotides known as tricyclo-DNAs (tcDNA) demonstrated unique pharmacological properties [20,21]. They were designed as analogues of bicyclo-DNAs, a first generation of conformationally constrained DNA analogues. In tcDNA, the C3′ and C5′ are connected by an ethylene bridge and fused to a cyclopropane ring (Figure 1) in order to limit torsional flexibility of the sugar backbone reducing the entropy of homoduplex and stabilizing heteroduplexes tcDNA/RNA. Fully modified tcDNA containing all four natural nucleobases show increases in thermal stability of duplexes with complementary DNA by ca 1.2 °C/mod and with complementary RNA by ca 2.4 °C/mod. TcDNA also revealed an average increase TM by about +1.2 °C/mod relative to 2′OME RNA ASO [22]. These tcDNA compounds are currently exclusively synthesised by SYNTHENA AG based in Bern, Switzerland.

As other ASO molecules, tcDNA phosphodiester backbone (PO-tcDNA) can be modified to its phosphorothioate version (PS-tcDNA). This chemical modification has been shown to increase stability and improve cellular uptake. Nevertheless, it is important to underline that tcDNA in their PO version are already resistant to nuclease activity compared to other phosphodiester based ASOs [11,22]. In this sense, phosphorothioate bonds are not needed to increase nuclease resistance although they were shown to enhance cellular uptake and biodistribution [23].

In this review, we will describe the various antisense approaches using tcDNAs and their preclinical advances, exploring the different applications of tcDNA with a particular focus on splice switching developments for Duchenne Muscular Dystrophy (DMD) and Spinal Muscular Atrophy (SMA) which are the most advanced preclinical programs. We will then discuss the advantages and potential limitations of the tcDNA chemistry, with a focus on the need for careful toxicological screen early in the process of ASO drug development.

## 2. Mechanisms of Action

**Figure 2:**
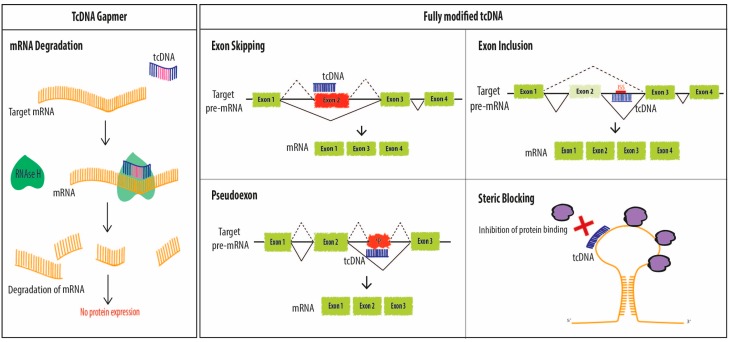
TcDNA-ASOs mechanisms of action. TcDNA, as other ASOs, may exert different effects depending on their structure and design. Fully modified tcDNA (right panel) are not able to elicit RNAse H activity and they are commonly used to manipulate alternative splicing (exon skipping, exon inclusion and correction of aberrant splice sites) or to prevent protein binding (steric blocking). However, in order to induce mRNA degradation via RNAse H, tcDNA must be designed as gapmers (left panel).

Antisense Oligonucleotides offer various therapeutic options including the original targeted gene knockdown, achieved through the recruitment of RNAse H to degrade mRNA at sites of DNA/RNA hybridization upon ASO binding. For this purpose, however, chemically modified ASOs need to be designed as “gapmers” with a natural DNA core (or “gap”) to induce RNAse H activity flanked by nuclease resistant-modified ends to improve drug stability and tissue distribution. Such chimeric oligonucleotides combine the features of high-RNA affinity and biostability while maintaining the ability to degrade the targeted mRNA via an RNase H mechanism (Figure 2). Fully modified ASOs on the other hand can be used for other applications aiming at the elimination of mRNA toxicity or the manipulation of alternative splicing. In this context, oligonucleotides also called SSOs can be used to modulate the ratio of splicing variants or correct splicing defects, by inducing exon-skipping or exon-inclusion, which opened far reaching implications in the treatment of a variety of diseases (Figure 2).

### 2.1. Splice Switching

Fully modified tcDNA can be used to correct splicing defects either by inducing exon-skipping, exon re-inclusion or masking aberrant splice sites. The first study demonstrating the potential of fully modified tcDNA to induce splice correction was performed on the human *β-globin* gene by Renneberg and colleagues in 2002 [22]. Authors used HeLa cell lines stably expressing the human *β-globin* gene with two different point mutations which induce inclusion of an aberrant exon in the mRNA. Transfection of a 17-mer tcDNA targeting the cryptic 3′-ss induced correction of the aberrant splicing with up to 100-fold enhanced efficiency relative to a 2′OME oligonucleotide of the same length and sequence. Moreover, tcDNA were able to mask aberrant splice sites in HeLa cells even in the absence of transfection agent, while 2′OME oligonucleotides were not capable of this.

#### 2.1.1. Exon-Skipping

Exon-skipping approaches using tcDNA were first performed on cyclophilin A pre-mRNA with the aim of inducing nonsense mediated decay and thus down-regulating cyclophilin A expression as a potential therapeutic inhibition of HIV replication [24]. In this study, tcDNA were compared to LNA, which also belong to the class of conformationnally constrained DNA analogue. ASO of different sequences were used to target the 3′ and 5′ splice sites of exon 4 of *cyclophilin A* in HeLa cells. The antisense efficacy of the tcDNA-ASOs was found to be superior to that of the LNA-ASOs in all cases by a factor of at least 4–5. Moreover, the strong exon skipping induced by tcDNA led to a reduction in cyclophilin A protein to 13% of its normal level. These results confirmed the potential of tcDNA for antisense application and their superiority compared to other oligonucleotides of the same class. Since then, exon skipping approaches using tcDNA have mostly focused on DMD where the ASOs are used to skip one or several exons in order to restore the reading frame.

DMD is a genetic, X-linked, muscle wasting disease which affects 1 boy in 3600. This disease is caused by different types of mutations in the *DMD* gene (deletions, duplications, insertions, point mutations) which mostly disrupt the open reading frame and thus lead to an absence of functional dystrophin protein [25]. Interestingly, Becker Muscular Dystrophy (BMD), which is also caused by mutations in the *dystrophin* gene, results in a much milder phenotype. In contrast to DMD mutations, BMD deletions do not disrupt the open reading frame giving rise to a partially truncated but functional dystrophin. The antisense-mediated exon-skipping for DMD aims to eliminate one or several exons, by masking key splicing sites with antisense sequences, to restore the reading frame and therefore induce the expression of a BMD-like dystrophin. This strategy should be applicable to a large proportion of patients (possibly up to approximately 83% of DMD patients) [26]. However, it is important to note that it will not be a definite cure and mostly aims at an improvement toward a BMD-like phenotype depending on the functionality of the restored protein.

In 1996, the first exon-skipping therapy for DMD was reported by Pramono et al. in lymphoblastoid cells [27]. Following these encouraging results, several in vivo studies have provided pre-clinical evidence for the therapeutic potential of an antisense strategy for DMD in various animal models. Especially the *mdx* mouse model, which carries a nonsense mutation in exon 23, has been used to test the efficacy of antisense approaches using different ASO chemistry such as 2′OME [28], phosphorodiamidate morpholino oligomer (PMO) [29,30], LNA and PNA [31,32]. Two of these chemistries have been evaluated in clinical trials and demonstrated encouraging results (drisapersen [33,34,35,36,37,38] and eteplirsen [38,39,40,41,42]). However, further clinical studies were not able to show a significant clinical benefit, probably due to the low levels of dystrophin restoration detected in DMD patients. While the US Food and Drug Administration (FDA) has approved eteplirsen, the clinical benefit still has to be demonstrated and additional clinical trials have been required.

Recently, we demonstrated the therapeutic potential of tcDNA (15-mers or 13-mers) in DMD mouse models following 12 weeks of systemic treatment [43,44]. TcDNA-ASOs were detected in all skeletal tissues as well as in the heart and the brain after intravenous injection and their long-lasting effect was measured as long as 12 weeks after the end of the treatment. Exon-skipping levels measured by quantitative PCR, were similar for 13-mers and 15-mers tcDNA but more importantly up to 5–6 fold higher than the levels achieved with 2′OME and PMO ASOs (chemistries of drisapersen and eteplirsen respectively). This treatment led to a far greater dystrophin restoration in tissues, especially in the diaphragm and the heart, where levels reached 50% and 40% respectively, compared to wild-type mice. Remarkably, tcDNA-ASO was the only chemistry able to induce exon-skipping and dystrophin protein expression in the CNS of treated animals, with a correct protein localization in stratum pyramidal and proximal stratum radiatum of CA1 hippocampus.

Furthermore, tcDNA treatment significantly improved muscle, respiratory and cardiac functions in *mdx* mice. Besides, thanks to the ability of tcDNA to restore dystrophin expression in the CNS, beneficial effects on *mdx* mouse behaviour were demonstrated. Tonic immobility (freezing) resulting from a restraint-induced fear response, controlled by central mechanisms, was returned to normal in tcDNA-ASO-treated *mdx* mice, as opposed to 2′OME and PMO ASO-treated mice. These studies confirmed the therapeutic potential of tcDNA for exon skipping mediated treatment of DMD and their superiority to other ASO chemistries.

#### 2.1.2. Exon Inclusion

Fully modified tcDNA-ASOs can also be used to promote exon-inclusion and a typical example is the re-inclusion of the exon 7 of the *SMN2* gene as a therapeutic strategy for the treatment of SMA.

SMA is an autosomal recessive disorder affecting 1 on 6000 birth and it is caused by mutations in the *SMN1* gene (*Survival Motor Neuron 1*) [45]. SMA is characterized by degeneration of α-motorneurons in the anterior horn of the spinal cord inducing progressive muscle weakness, paralysis and often death. In humans, the severity of the disease is lessened by the presence of *SMN2* (a centromeric copy of *SMN1*) [46]. These two genes differs by only a C to T in the exon 7 coding sequence leading to the exclusion of exon 7 in 90% of mature transcripts [47,48]. As a result, SMA patients have only a few SMN protein encoded by the *SMN2* gene. Moreover, SMA disease displays 4 degrees of severity which are correlating with the copy number of *SMN2* gene [49]. These findings highlight the therapeutic potential of exon 7 re-inclusion in SMN2 mRNA using ASOs and several research groups have investigated this approach. Some of the most encouraging results were obtained with 2′-*O*-methoxyethyl (2′MOE) where intracerebroventricular injections (ICV) induced phenotypic rescue of type III SMA mice and increased survival of type I SMA mice following subcutaneous injection [50,51,52]. These preclinical data led to clinical evaluations in SMA patients and recent approval of nusinersen by the FDA [53,54]. 

Since 2′MOE cannot cross the blood brain barrier, nusinersen is administered directly into the spinal fluid of patients by intrathecal injection. As an alternative, we recently evaluated tcDNA for the treatment of SMA [55]. TcDNA-ASOs targeting the ISS-N1 previously described [56] were first tested in vitro in SMA type I fibroblasts in which they could increase the level of exon 7 inclusion to 64%. Moreover, the SMN protein was increased to up to 90% of wild-type levels, correctly localized in nuclear gems and the number of gem-positive nuclei was restored to the level found in fibroblasts of healthy individuals [55]. Weekly subcutaneous injections of tcDNA-ASOs in SMA type III mice achieved up to 55% of exon 7 inclusion after 4 weeks of treatment and up to 75% after 12 weeks in skeletal muscle. Moreover, after 12 weeks of treatment, exon 7 inclusion was increased by 1.3 fold in the brain confirming the ability of tcDNA to cross the BBB [55]. Importantly, tcDNA treatment was shown to rescue the phenotype of SMA type III mice which typically display progressive necrosis of tails, ears and toes. In addition, some parameters of respiratory function of treated mice evaluated by plethysmography, was restored to wild-type levels suggesting that an increase of exon 7 inclusion to 60% in the diaphragm is correlated with an improvement in respiratory function [55].

### 2.2. Steric Blocking Applications

#### 2.2.1. Inhibition of *Trans*-Activation

Antisense oligonucleotides can also be used as steric blocking inhibitors and one of this example is their potential application related to Human Immunodeficiency Virus type-1 (HIV-1) disease.

The transcription of the HIV-1 genome is activated by mechanism that involves the interaction of the viral protein Tat with the *trans*-activation responsive element (TAR) RNA [57]. ASOs that are complementary to the region of TAR RNA are obviously good alternative inhibitors of Tat-mediated *trans*-activation [18,58,59]. Steric blocking ASOs were used in that way to inhibit viral replication [60] and different chemistries of ASOs were previously tested including 2′OME, mixmers of 2′OME and LNA, or PNA [61,62,63,64]. These molecules induced the inhibition of Tat-dependent in vitro transcription by the prevention of HIV-1 Tat binding to TAR RNA.

Ivanova and colleagues showed that tcDNA-ASOs could also be used to inhibit the Tat-dependent *trans*-activation in HeLA cells. Although tcDNA oligonucleotides bound TAR RNA more weakly, they were as good as 2′OME/LNA oligonucleotides in suppressing in vitro transcription and *trans-*activation in HeLa cells, confirming their efficacy for such steric blocking approaches [65].

#### 2.2.2. Reduction of mRNA Induced Toxicity

Antisense oligonucleotides have shown high efficacy in reducing the RNA toxicity due to triplet expansion in multi systemic diseases such as myotonic dystrophy type I (DM1). DM1 is an autosomal dominant inherited disease and the most represented adult-onset form of muscular dystrophy with a prevalence of 1 to 8000 people over the world [66]. Patients suffering from DM1 show muscle weakness, myotonia, insulin resistance, cardiac failure, smooth muscle dysfunction and neurological abnormalities [67]. DM1 is caused by expanded CTG repetition in the untranslated 3′UTR region of the Dystrophia Myotonica Protein Kinase (DMPK) [66,68,69]. Patients with DM1 present CTG repeat lengths of 50 to several thousands, when healthy people carry less than 38 repeats in this region [67].

The presence of CUG repeats expansion in the RNA of an unrelated gene (*human skeletal α-actin*, HSA) has also been shown to induce similar clinical signs of the myotonic dystrophy [70], confirming that the pathogenesis of the disease is predominately related to their RNA toxic effect. Several studies have been conducted to understand the underlying processes which contribute to the toxic effect of the expansions within the RNA and principally two mechanisms were described. First, CUG repeats expansion in the RNA can form double stranded hairpin structures. These structures are able to interact and link the Muscleblind like 1 (MBLN1) proteins, leading to its sequestration [71,72]. The second described mechanism is the activation of the protein kinase C (PKC), which leads to the hyperphosphorylation and upregulation of a splicing factor (CELF1) [73,74]. The loss of function of MBLN1 and the gain of function of CELF1 contribute to the pathogenesis of DM1 by inducing aberrant alternative splice events such as the chloride channel (Clcn1), the insulin receptor (IR) and the bridging integrator 1 (BIN1) [75,76,77].

The inhibition or the reduction of the RNA toxicity, targeting the CUG triplet expansions represent a valuable therapeutic strategy for myotonic dystrophy. The rationale of this therapeutic approach is to design antisense molecules targeting the repetitions to block the toxic effect. Different studies have been conducted using this strategy with different ASOs chemistries: a PMO sequence CAG25, a 2′OME CAG7 and a peptide conjugated-PMO (PPMO) which have all showed their efficacy for DM1 [78,79,80]. These molecules bind the pre-mRNA and prevent the binding and the sequestration of the protein MBLN1, which therefore leads to the reduction of the toxicity and the correction of the alternative splicing of the target genes.

Similar preliminary results have been achieved using fully modified tcDNA-ASOs targeting the CUG repeats, showing significant reduction of the RNA toxicity in DM1 and subsequent rescue of alternative splicing (Personal communication D. Furling and colleagues). These studies provide evidence that the reduction/inhibition of the RNA toxicity using ASOs targeting the CUG repeat expansions is an effective therapeutic approach for treating the physiopathology of DM1 patients.

### 2.3. Degradation of mRNA

Like other ASO chemistries, tcDNA can also be used for targeted gene knockdown, achieved through the recruitment of RNAse H upon ASO binding. To this end, tcDNA-ASOs must be designed as gapmers, composed of natural nucleotides in the centre of the sequence flanked by chemically modified wings on either side to protect them from nucleases degradation (Figure 2). Murray and colleagues investigated the use of tcDNA oligonucleotides as gapmers in comparison with their 2′-*O*-methoxyethyl (2′MOE) and 2′,4′-constrained ethyl (cEt) counterparts targeted against the scavenger receptor B1 (SR-B1) [23]. Different lengths of tcDNA gapmers were designed (20, 18, 16 and 14-mer) and compared to a 20-mer 2′MOE and 14-mer cEt in cell culture and animal experiments. The 20-mer tcDNA gapmer was found with excellent activity for reducing the SR-B1 mRNA in liver and showed better activity in several extra-hepatic tissues such as kidney, heart, diaphragm, lung fat and skeletal muscles. This study provided an impetus to further explore tcDNA-ASOs for therapeutic antisense applications using the RNase H mediated degradation.

Gapmer ASOs have been used to down-regulate the expression of many genes and thus considered promising therapeutics for numerous diseases such as Huntington’s Disease. Huntington’s Disease (HD) is a neurodegenerative condition characterized by locomotor, cognitive and behavioural disorder [81]. HD is an autosomal dominant disease caused by the expansion of CAG repeat in the *huntingtin* gene and affect 5–6 Caucasians in 100,000 [82]. These expansions of CAG repeats lead to an expanded polyglutamine mutated protein which particularly aggregates and have toxic effects on neurons [83]. While the precise roles of the Huntingtin protein (HTT) and its mutated version are still debated, therapeutic strategies aiming at down-regulating one or both are currently being investigated. Amongst the antisense approaches available for HD, one of the most advanced approaches is the use of a 2′MOE gapmer targeting the exon 36 of human *HTT* and inducing *HTT* silencing by the activation of RNase H developed by IONIS pharmaceuticals. Intracerebroventricular injection (ICV) of this 2′MOE gapmer in mice for 2 weeks at 50 µg/day significantly decreased the level of mRNA and protein with a persistent effect for 12 weeks after the end of the treatment [84]. Moreover, 9 months after the end of treatment phenotypic amelioration can still be detected indicating that the beneficial effect persists longer than the silencing [84]. Injections of this molecule in non-human primates was well tolerated and revealed a large biodistribution of the drug in the different CNS compartments. These encouraging pre-clinical results have led to the evaluation of this 2′MOE gapmer (IONIS HTTRx) in clinical trials in which the drug is directly injected in the spinal fluid (intrathecal administration) of HD patients [84]. Recent press release revealed that the safety and tolerability profile of IONIS-HTTRx in the completed cohorts of the Phase 1/2a study supported its continued development and that an ‘open-label extension’ will be activated for the volunteers in the current trial.

Considering the interesting properties of tcDNA-ASOs, in particular regarding their biodistribution and ability to cross the blood brain barrier, we have recently started investigating the therapeutic potential of tcDNA gapmers for HD. Transfection of different HD patient fibroblast cell-lines has shown that tcDNA gapmers reduce significantly the level of both mutated and wild-type mRNA and protein (respectively 70% and 30% of protein reduction) and this more efficiently than 2′OME ASO used in parallel. Single ICV injections of tcDNA gapmer in YAC128 mice carrying wild-type murine *HTT* and human mutated *HTT*, induce 40 to 60% reduction of mRNA and mutated protein 2 weeks after the treatment in various CNS compartments. This decrease appears even more important 6 weeks after the treatment and the effect of the treatment was found to persist for at least 12 weeks [85](Imbert et al. manuscript in preparation). These results indicate that tcDNA gapmers targeted against the HTT mRNA are well tolerated in mice and represent an alternative ASO chemistry for the treatment of HD. Besides, since tcDNA-ASOs are able to cross the BBB, systemic treatment could be envisaged allowing the treatment of tissues and organs outside the CNS which might substantially improve the quality of life of patients with Huntington’s disease, even in the absence of disease-modifying effects [86].

## 3. Properties and Advantages

Throughout the various applications reviewed above, tcDNA-ASOs clearly appear as promising ASO alternatives with high therapeutic potential and unique properties. One of the most outstanding one is their ability to cross the BBB at low levels after systemic administration such as intravenous or subcutaneous delivery. This property, demonstrated in various mouse models (*mdx*, dKO, SMA and WT mice) opens new therapeutic avenue for neuromuscular and neurological disorders. Because of the inability of most ASOs to cross the BBB, current antisense treatment for disorders like HD or SMA (whether being in trials or already approved) have to be administered via the intrathecal or ICV route. Despite encouraging data and positive outcomes reported in patients so far, these ways of administration present obvious clinical challenges. Moreover, they neglect the issue of delivering ASOs to the periphery, which could be crucial for SMA or HD [86]. In these context, tcDNA-ASOs could deal with the issue of both peripheral and central tissue penetration but also provide a simpler mode of administration which is more easily administered and better tolerated. The advantageous uptake properties of tcDNA are not limited to the CNS and we have identified many other target tissues of phosphorothioate tcDNA-ASOs after intravenous administration, including retina, smooth muscles, fat and skin, which opens up even more foreseeable therapeutic applications.

The mechanisms responsible for the enhanced activity of tcDNA are not yet fully characterised. We have previously described that tcDNA-ASOs were spontaneously forming defined nanoparticles ranging from 40–100 nm using nanoparticle tracking analysis (NTA), Size-Exclusion Chromatography with Multi-Angle Static Light Scattering (SEC-MALS) and dynamic light scattering (DLS) [43,87]. This propensity to spontaneously self-assemble into nanoparticles could imitate transfection reagents and nanoparticle delivery systems [88] and hence could potentially explain improved cellular uptake. The mechanism of spontaneous tcDNA self-assembly is still not well understood. This could be partly due to their increased hydrophobicity which might confer secondary amphipathic properties on the structure permitting self-assembly similar to amphipathic peptides [89]. It can also be hypothesized that the tcDNA extra rings facilitate the generation of poly-G-like structures that have been seen to enhance the uptake of ASO and their binding to scavenger receptors [90].

One of the main attractive features of tcDNA-ASOs is clearly their higher RNA binding affinity, permitting use of ASOs of decreased length. As mentioned previously, we recently demonstrated that systemic delivery of 13nt-tcDNA allows similar exon skipping levels in skeletal muscles than a 15nt, with even higher levels in the heart. Importantly, this characteristic offers the advantage of reducing the mass of synthetic nucleotides administered, which could decrease the toxicity induced by accumulation of ASO.

Each class of ASO from first to third generation has shown stereotypic toxicity profiles and these nuances are important for the toxicological pathologist to be aware of [91]. Evaluation of toxicology is particularly important when developing new generation of ASOs to avoid subsequent failure of a new drug in further toxicological studies. We have recently evaluated the toxicological profile of tcDNA-ASOs in DMD mouse model and shown that high dose tcDNA treatment (200 mg/kg/wk for 12 weeks) was well-tolerated in all mice [44]. Because of the well-known immunostimulatory effects of PS ASOs [92,93], complement activation and cytokines levels were evaluated. No significant differences were observed on C3 protein or cytokines levels in *mdx* treated mice compared to *mdx* control mice. Antisense molecules are known to accumulate in some tissues and induce additional adverse effects such as liver and kidney injury, especially in repeated-dose studies. However, no significant changes were noted in serum biomarkers compared to untreated *mdx* mice. Histological findings were limited to minimal glomerular changes and few cell necrosis in proximal tubules and minimal liver inflammation. To investigate the toxicological profile of tcDNA more deeply, some early biomarkers of renal toxicity were measured [94] and a significant, albeit moderate upregulation was detected in several biomarkers (KIM1, renin, urinary albumin), indicating some renal toxicity due to tcDNA accumulation in kidney tubular cells. Similar changes were previously reported with 2′OME PS-ASO, confirming the expected effect of PS-ASO accumulation in kidneys [95]. Importantly, no unexpected class-related toxicological issues emerged following tcDNA treatment.

These results demonstrated an overall encouraging safety profile but yet highlighted the well-known issue of PS-ASO accumulation and associated kidney toxicity. To circumvent this issue, we recently investigated the effect of phosphorothioate linkage content within tcDNA backbone on ASO efficacy and toxicity. As mentioned in the introduction, it is important to underline that tcDNA in their PO version are already resistant to nuclease activity compared to other phosphodiester based ASOs [11,22], so the phosphorothioate bonds are not required for nuclease resistance but mainly to enhance cellular uptake and biodistribution [23]. Our results confirm that the amount of PS linkages correlates with some level of toxicity and that it could be reduced by decreasing the number of PS content without affecting their efficacy on exon skipping or dystrophin restoration [96] (Echevarr*í*a et al. manuscript in preparation). An optimised balance between PO and PS bonds can therefore be achieved to induce maximal efficacy without the PS associated side effects. However full reglementary toxicological studies remain to be completed to insure the clinical translation of this promising ASO chemistry and its safety in patients. 

## 4. Conclusions

Despite major advances in chemistry and design, the challenge of ASO delivery remains one of the largest obstacle in the antisense field. International efforts are currently focusing on new generations of ASOs or different drug delivery systems such as various conjugations to find the best clinical candidates in term of efficacy and safety profile. Amongst these new promising chemistries, tcDNA have shown interesting properties and clear superiority compared to some clinically tested ASOs, which is partly due to their higher RNA binding affinity and increased hydrophobicity. The properties of tcDNA described in this review make them particularly attractive as ASOs drugs for many genetic diseases. Clinical evaluation of tcDNA for the treatment of DMD is currently being planned but like all other chemistries, the outcome still crucially depends on how well it will be tolerated in humans.

Within the last few years the prospect of successful ASO-based splicing therapy for neuromuscular disorders has become a reality, with antisense compounds recently approved for DMD and SMA. Continuous attempts to ameliorate ASOs pharmacological properties in order to increase their tissue distribution and cellular uptake using further chemical modifications or conjugation to various ligands contribute to advance the therapeutic potential of ASOs. There is no doubt that such optimisation of the tcDNA chemistry will lead to second and third generation compounds with even more potent properties for the treatment of genetic diseases.
